# Uncovering the Industrial Potentials of Lemongrass Essential Oil as a Food Preservative: A Review

**DOI:** 10.3390/antiox11040720

**Published:** 2022-04-06

**Authors:** Fatima Faheem, Zhi Wei Liu, Roshina Rabail, Iahtisham-Ul Haq, Maryam Gul, Marcin Bryła, Marek Roszko, Marek Kieliszek, Ahmad Din, Rana Muhammad Aadil

**Affiliations:** 1 National Institute of Food Science and Technology, University of Agriculture, Faisalabad 38000, Pakistan; fatimafaheem538@gmail.com (F.F.); roshina.rabail@gmail.com (R.R.); maryamgul1608@gmail.com (M.G.); ahmad.din@uaf.edu.pk (A.D.); 2 College of Food Science and Technology, Hunan Agricultural University, Changsha 410128, China; zwliu@hunau.edu.cn; 3 Kauser Abdulla Malik School of Life Sciences, Forman Christian College (A Chartered University), Lahore 54600, Pakistan; iahtisham@fccollege.edu.pk; 4 Department of Food Safety and Chemical Analysis, Prof. Waclaw Dabrowski Institute of Agricultural and Food Biotechnology—State Research Institute, Rakowiecka 36, 02-532 Warsaw, Poland; marcin.bryla@ibprs.pl (M.B.); marek.roszko@ibprs.pl (M.R.); 5 Department of Food Biotechnology and Microbiology, Institute of Food Sciences, Warsaw University of Life Sciences—SGGW, Nowoursynowska 159 C, 02-776 Warsaw, Poland

**Keywords:** lemongrass, antimicrobial, antioxidant, food preservative, novelties

## Abstract

The food industry is growing vastly, with an increasing number of food products and the demand of consumers to have safe and pathogen-free food with an extended shelf life for consumption. It is critical to have food safe from pathogenic bacteria, fungi, and unpleasant odors or tastes so that the food may not cause any health risks to consumers. Currently, the direction of food industry has been shifting from synthetically produced preservatives to natural preservatives to lower the unnecessary chemical burden on health. Many new technologies are working on natural prevention tools against food degradation. Lemongrass is one such natural preservative that possesses significant antimicrobial and antioxidant activity. The essential oil of lemongrass contains a series of terpenes that are responsible for these activities. These properties make lemongrass acceptable in the food industry and may fulfill consumer demands. This article provides detailed information about the role of lemongrass and its essential oil in food preservation. The outcomes of the research on lemongrass offer room for its new technological applications in food preservation.

## 1. Introduction

Food products are abundant in nutrients that bring in bacterial and fungal colonizers along with other pests such as insects and rodents. Bacteria, fungi, and yeasts grow well in environmental conditions that support their growth. For example, food products rich in carbohydrate content with low pH and low water content do not support spoilage due to bacterial activity because such conditions do not promote their growth. However, perishable, highly perishable and unpasteurized foods, with neutral or alkaline pH levels, provide a favorable environment to yeast and molds that leads to the deterioration of food products [1]. Between 2016 and 2018, the US Food and Drug Administration (FDA), Centers for Disease Control and Prevention (CDC), and state and local officials investigated several multistate outbreaks involving fresh-cut products of vegetables and fruits consumed as salads [2]. Many vegetables also lose their freshness when exposed. For example, fresh-cut potatoes are sensitive to microbial deterioration and suffer from quick quality loss due to their high moisture content and high metabolic rate [3,4]. While talking about food deterioration caused by pathogens, meat is most dominant due to its high susceptibility. In meat, microbes cause changes in pH, the formation of slime, and organoleptic changes with foul smell [5].

Microbes that cause food spoilage enter the food through different routes regardless of the processing and storing techniques. For many microorganisms, fresh fruit and vegetables provide a suitable environment for growth and survival [6]. In every category of food material, *Klebsiella, Bacillus,* and *Pseudomonas* are the main responsible genera for degradation. *Klebsiella* is a bacterium that is found to be effective at causing spoilage in meat, fruits, and vegetables. In addition, most of the spoilage problems are caused by *Pseudomonas* species in dairy and fatty products [7]. From many spoilage samples, *Staphylococcus* spp., *Micrococcus* species, and *Escherichia coli* are also retrieved [8]. Furthermore, it is found that species of *Acidovorax, Shigella, Listeria, Salmonella,* and *Xanthomonas* bring about decay in fruits [7].

Moreover, fungi and molds are both involved in the spoilage of processed products and fresh products. Fungal species such as *Pythium, Phoma, Penicillium, Aspergillus, Alternaria, Fusarium, Paecilomyces, Rhizopus,* and species of mold such as *Ceratocystis, Rhizoctonia,* and *Botrytis* are known to cause deterioration [7]. The yeast species *Saccharomyces cerevisiae* is found to have a potential effect on the spoilage of fruit juice [9]. Fungal species such as *Fusarium*, *Penicillium, Alternaria, Macrophomina, and Aspergillus* have the potential to cause damage to sorghum, rye, wheat, cowpeas, rice, maize, groundnuts. Fungi mostly grow in fruits and vegetables as they are rich in nutrients and have an optimum pH for fungal growth, while their acidity acts as a barrier for some spoilage-inducing bacteria. Spoilage-inducing organisms infest their hosts by crossing their natural protection of the cell wall barrier by creating extracellular lytic enzymes that degrade the host body; for example, hemicelluloses and pectinases are lytic enzymes generated by fungi [7].

After invasion, microorganisms start the process of colonization. Colonization of fungi occurs through the accumulation of conidia on the product, which is at first in its inactive form (vegetative), but eventually becomes converted into the active form (reproductive) and starts germination. This conversion is supported by various factors, including nutrients like glucose, phosphate, temperature, osmolarity, and water content. The germination of fungi leads to the production of harmful fungi, which may result in the production of mycotoxins that are toxic to food and health. After invasion and infection, bacteria produce unpleasant organoleptic changes in the taste, smell, and colour of the food product [10,11]. In addition to microbes, insects also have the potency to cause damage to post-harvest food products. They can reduce the quality of grains and fruits. Besides insects and microbes, other factors include the oxidation of food products and enzymatic activities; these are natural processes, but they can result in rancidity which ultimately can reduce food’s acceptability due to unpleasant odors and tastes.

Many recent studies have turned their main focus towards this critical issue of food quality enhancement, but still, there is a gap in the identification of naturally gifted food sources with preservative qualities therein, along with their novelties in their utilization. Therefore, in this review, an attempt has been made to highlight such innovations.

## 2. Use of Natural Plants as Preservatives

Researchers have investigated alternate non-thermal processing techniques for developing shelf-stable foods with clean label technology [3,12,13,14]. From many years, including presently and potentially in the future, the use of spices and herbs to enhance the taste, appearance, and fragrance of food products has been utilized, and it remains a preservative practice that mankind actively engages in. In the modern era, the essential oils of these spices and herbs have been gaining much recognition regarding their utility against the microbial spoilage of food and associated possible health problems [15]. These bactericidal, fungicidal, insecticidal, anti-spasmodic, anti-inflammatory, anesthetic, and tranquilizing properties of herbs and their essential oils have earned them a place in food preservation technology [16,17].

Food deterioration is a phenomenon that causes huge losses to economics, and the ingestion of spoiled food can cause many serious illnesses. For instance, it has been observed that food can be spoiled due to the presence of enzymes and lipids. This presence can cause lipids’ self-oxidation and produce an unpleasant odor and taste. These changes primarily occur in meat and dairy products [18,19]. To prevent health hazards, reduce economic losses, and preserve food, the use of synthetically produced chemicals is common to kill microbes. It has been noted by health agencies like the Food and Agriculture Association (FAO) that synthetically produced antimicrobial agents cause serious health problems; therefore, the use of those chemicals has been restricted. The inhibition of such chemicals has led to other sources that can be used instead. Therefore, the use of naturally occurring food preservatives is a positive approach that has drawn the attention of food industries and led to much research. For that, the term essential oil comes into existence. Essential oils are the secondary metabolites of plants, and they possess a desirable taste. Though plant oils have been used in enhancing taste, they have extensive use in the preservation of food products [17].

Food needs to be preserved, safe from microbes, should have consumer acceptability with a prolonged shelf life, and should be free from health hazards. The food industry is working to fulfill these demands, but it is very challenging for them because of an increasing number of food products that require a longer shelf life. The use of lemongrass essential oil (LEO) may overcome all the challenges that are faced by the food industry. This article deals with the preservative properties of LEO and the mechanism behind it. This article also contains information regarding technologies that have been introduced to make LEO effective for preservation.

## 3. LEO as a Preservative

**Figure 1:**
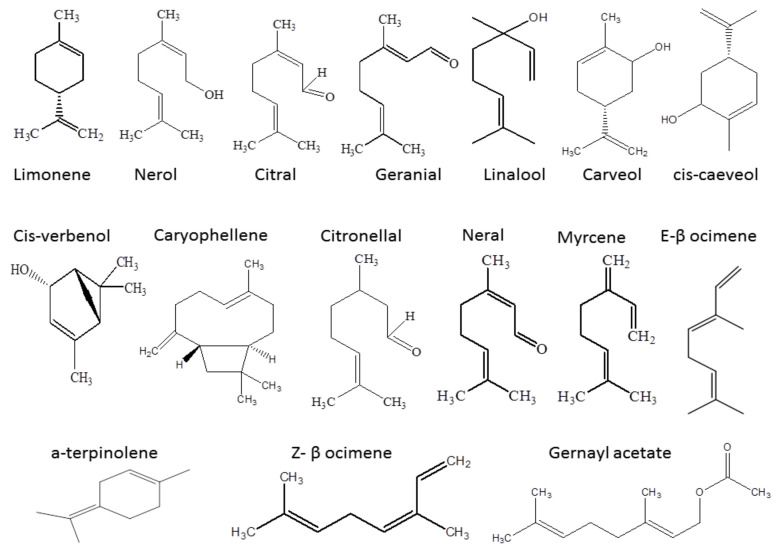
Bioactive components extracted from LEO.

*Cymbopogon* (Lemongrass) species are cultivated in Central and South America, semitropical and tropical areas of Asia, Africa, and other tropical countries. There are about 55 species. Among them, *C. ambiguous*, *C. ombicynus, C. obtectus, C. refractus,* and *C. procerus* are found in Australia, *C. citratus* is found in China, *C. citriodora* is found in West India, *C. flexuosus* is found in East India, *C. nardus* is found in Thailand, *C. proximus* is found in Egypt, and *C. schoenanthus* is found in North Africa and South Asia [10]. Lemongrass has a non-toxic mechanism of action and is thought to be safe for both human health and the environment. Lemongrass oil, a prominent culinary component in many cuisines, is non-toxic to humans and most non-target species. LEO is a food additive that is Generally Recognized As Safe (GRAS). LEO is registered under the REACH (Registration, Evaluation, Authorization and Restriction of Chemicals) regulation by the European Chemical Agency (ECHA) [20]. An essential oil is defined as an ‘aromatic raw materials’ odorous product, usually of complex composition, obtained from plants’ raw material by steam distillation, dry distillation, or a suitable mechanical process without heating’ by the European Pharmacopoeia and the International Standard Organisation (ISO) ISO 9235:2013/Cor 1:2014. Of the estimated 3000 essential oils identified, roughly 150 are commercially important and sold on global markets today [21]. Among these essential oils, LEO protects the sensory properties of food and hinders the activity of microbes, preventing food deterioration, preserving the product quality and extending the shelf life of food [22]. Likewise, this preservative benefit of LEO can be attributed to its active component citral, which constitutes the major component of LEO (>45%) and has a unique lime aroma. Though citral is present in lemongrass, it is now being produced synthetically [23]. The chemical structure of various bioactive components present in LEO is presented in Figure 1.

A widely used species is *C. citratus*, the essential oil of which consists of geranial and neral, with two geometric isomers, known as citral a and citral b, respectively, in its oil. It is reported that 1.85% essential oil of lemongrass is obtained using solvent extraction, and 0.86% is yielded by the steam distillation method from lemongrass leaves [24]. Citral, which is an aldehyde, gives a strong lemony odor to *C. citratus*. The essential oil that is extracted from *Cymbopogon* species, in addition to citral, contains myrcene, limonene, E, E-cosmene, Z-β-ocimene, E-β-ocimene, α-terpinolene, cis-verbenol, citronellal, linalool, cis-carveol, nerol, atrimesol, geranial, carveol, geranyl acetate, and caryophyllene. The amount of citral determines the quality of the lemongrass [25]. LEO is phototoxic in addition to cytotoxic. Phototoxicity indicates that when cells are subjected to light, the specific molecules of essential oil residing in the cells get excited and cause a transmission of energy that results in the production of a single oxygen. That production leads to the destruction of macromolecules, i.e., lipids, proteins, and DNA, of the cells [26]. At the cellular level, essential oils act in a variety of ways to modulate phototoxicity. These include the suppression of photosynthetic activity and mitochondrial respiration, microtubule disturbance and genotoxicity, regulation of phytohormones and enzymes, alteration of water status, membrane properties and their interrelations, and reactive oxygen species induction [27]. Although many other essential oils may also possess bioactive components therein, due to the presence of these bioactive components with great preservative potential, LEO is selected for a detailed overview here in this study.

### 3.1. Antibacterial Activity of LEO

Essential oils held great importance as preservatives against bacterial species. Among oils, LEO is a potent preservative. It is effective on many bacterial species and genera when compared with other essential oils such as *Listeria monocytogenes*, *Yersinia, Escherichia coli, Staphylococcus, Salmonella* Typhimurium, *Lactiplantibacillus plantarum, Pseudomonas aeruginosa, Bacillus cereus, Proteus vulgaris, Bacillus subtilis, Enterococcus faecalis*, and *Enterobacter aerogenes*, as elaborated from various studies in Table 1. The antimicrobial effect of LEO is more efficient on Gram-positive than Gram-negative bacteria. When bacteria such as *Listeria. welshimeri* and *Listeria monocytogenes* were tested, the result revealed that LEO was more effective than garlic essential oil and had a lower minimum bactericidal concentration and minimum inhibitory concentration. Gram-negative bacteria are resistant due to the presence of an extra layer on their outer membrane, which is composed of hydrophilic lipopolysaccharides. The presence of this layer acts as an impervious hindrance, although the essential oils have a lipophilic nature [28].

**Figure 2:**
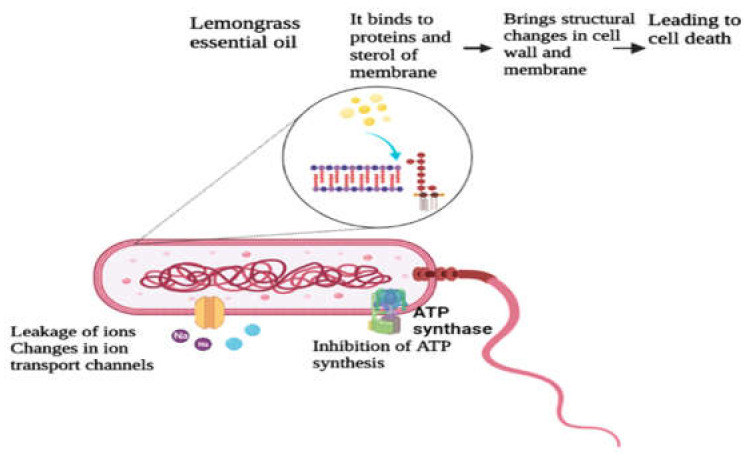
Effect of lemongrass essential oil on the bacterial cell.

Species of lemongrass contain terpenoid constituents such as citral, α-terpinene, geranial, linalool, neral, α-pinene, myrcene, and γ-terpinene. These terpenes are effective in the degradation of bacteria, as these are cytotoxic and cause toxic effects in the cell membrane and cytoplasm. These effects result in cellular degradation, loss of enzymes, phospholipid bilayer breakdown and damage to the genetic material. This overall antibacterial activity of LEO causes cellular lysis, structural changes, formation of spheroplasts, inhibition of the formation of a septum, and production of de-shaped cells. This further results in the destruction of proteins of the membrane, inhibition of ATP production, increased membrane permeability, leakage of ions, and changes in ion transport channels [45]. Figure 2 explains this overall cellular degradation of bacterial cells by LEO. Therefore, lemongrass and its essential oil are beneficial in preserving food such as juices, grains, raw meat, and bakery goods by offering better quality and increasing their shelf life.

### 3.2. Antifungal Activity of Lemongrass Essential Oil

Gliotoxin, ochratoxin A, fumitoxins, and malformins are mycotoxins produced by the species of Aspergillus that are cancer-causing in living organisms. The toxins produced by fungi are known to pose some danger to health, including the risk of cancer, risk of birth defects, toxicity to the kidneys, brain, and liver, suppression of immunity, and Kashin–Beck disease, which is the necrosis (death) of the growth plates of bones and joint cartilage [46]. Alongside its bacterial activity, lemongrass plays a vital role against fungus, aflatoxins, molds, and yeasts [47]. It has been noted that lemongrass is more effective when used in the vapor phase than in the liquid phase. According to the study by Reyes-Jurado, et al. [48], the possible mechanism behind the effectiveness of the vapor phase is that the micelle formation of lipophilic molecules occurs in the liquid phase, which hinders the connection between essential oil and organisms [48]. LEO possesses promising activity against *Candida albicans*, *Aspergillus niger*, and *C. tropicalis*, with the most significant activity against *Candida* species in the vapor phase [49]. According to the study by Oliveira, et al., LEO showed inhibition of mycelial growth and sporulation of *Aspergillus* species in oats (Table 1). Food deterioration that is caused by yeast species such as *S. cerevisiae*, *S. uvarum*, and *C. oleophila* and fungi such as *Penicillium roqueforti*, *Penicillium corylophilum*, *Eurotium repens*, *Aspergillus flavus*, *Endomyces fibuliger*, *A. alternata*, and *Fusarium solani* is controlled or inhibited by LEO [32,50,51].

**Figure 3:**
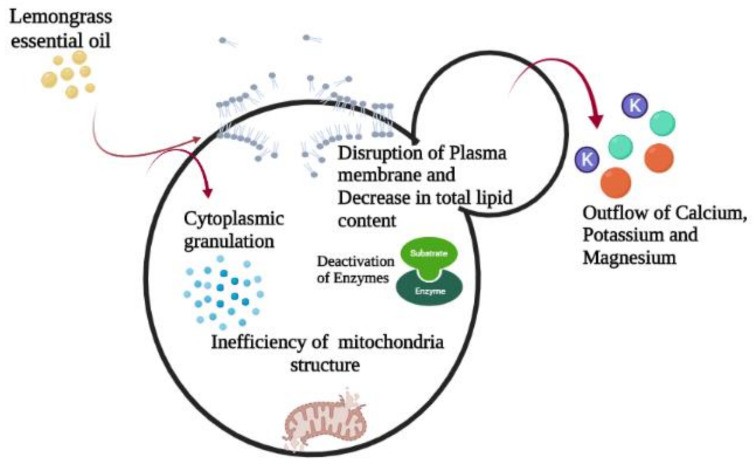
Effect of lemongrass essential oil on a fungal cell.

It has been reported that the cell membrane of the fungi is a possible target for essential oils to perform their function. The transportation of ions and nutrients across the membrane, the potentiality of the membrane, and the penetrability of the membrane of fungal cells are affected by the actions of essential oils on membranes. The changes that occur in fungal cells cause the death of fungi even in the presence of a promising environment [52]. In addition, there is also an outflow of calcium, potassium, and magnesium ions in disinfected mycelia. Moreover, the amount of unsaturated and saturated fatty acids upsurges, whereas there is a decrease in the total lipid content. There is also an outflow of ions, but that does not indicate the absolute failure of the function of the membrane, as this process also occurs in viable cells that are showing growth inhibition. This happens because the cell consumes energy for its repair and survival instead of propagation. This whole process affects the germination of conidia due to the loss of ions, which indirectly causes changes in the cell membrane, as shown in Figure 3. The fungal pathogens become static and latent when treated with LEO because it causes the inhibition of fungal growth and propagation without destroying the fungi [51]. LEO causes the inhibition of aflatoxins (mycotoxin) by not only the inhibition of germination but also by the production of the aflatoxins fumonisin, G2, G1, B1, and B2 [53].

There are three levels on which the inhibition of biosynthesis of aflatoxin takes place. (1) Alterations in physiological and surrounding components affect the biosynthesis of aflatoxin. (2) Signaling circuits that carry out the biosynthetic pathways are inhibited. (3) The expression of genes and activity of enzymes are directly inhibited in the pathway. There are many inhibitory compounds present in lemongrass that affect the transcription of a gene or particular steps of enzyme activity in the biosynthetic pathway, but their mode of action is still unknown. It was concluded that the physiological and environmental modulators important in the biosynthesis of aflatoxins and membrane signaling pathways are altered by the active constituents of lemongrass. Though much research has been done related to the inhibition of the three steps of the biosynthesis pathway of aflatoxins, including the oxygenation and peroxidation of lipid by the anti-mycotoxin activities of many essential oils [50], further research on LEO still needs to be done.

Essential oils cause the depolarization of mitochondrial activity in eukaryotic cells, affecting ionic calcium flux and other ionic pathways, lowering the pH gradient, and influencing proton pumps and the ATP pool. Following the release of free radicals, cytochrome C, and Ca^2+^, the chain reactions from the cell wall or cell membrane attack complete cells, causing severe oxidative damage and bio-energetic malfunction. Furthermore, cell death occurs when the outer and inner mitochondrial membranes permeabilize [54].

Myrcene, geranial, geraniol, linalool, citral, limonene, caryophyllene, eugenol, neral, and geranyl acetate are integrated and mutually perform the antimycotic food preserving function of this plant, having medicinal properties according to empirical investigations [55,56,57]. Linalool, for instance, has been shown to hinder spore germination and fungal development; the inhibition of sporulation is thought to be related to inhibition of the respiratory system of aerial mycelia. However, according to a study, geraniol’s mechanism of action involves an increase in potassium release outside the cell [58]. Geraniol extracted from lemongrass has shown maximum activity against microorganisms [59]. Citral, on the other hand, has been found to assault target species’ microtubules and has other cell-death-causing properties [60].

### 3.3. Antioxidant Activity of Lemongrass Essential Oil

Oxidation is the process in which free radicals are produced that destroy the quality of food products, resulting in the production of unpleasant odors and off-flavors. To ensure the quality of food products, antioxidants are used to preserve the food. People nowadays are conscious about using bio-preservatives when their health is concerned. For that matter, essential oils are being used to tackle oxidation in food products, so LEO holds importance as a bio-preservative that not only possesses antimicrobial activity but is also a potent antioxidant. The efficacy of bio-preservatives depends upon their antioxidant activity. Lemongrass contains monoterpenoid components (myrcene, eugenol, β-citral, and α-citral) that demonstrate antioxidant activity, especially eugenol, which contains scavenging properties against free radicals. It has been shown that lemongrass possesses the same antioxidant potential as that of BHT (butylated hydroxytoluene), which is synthetic. The methods that are used to check the antioxidant activity of Indian lemongrass include the 2,2-diphenyl-1-picrylhydrazyl (DPPH) method, reducing power assay, carotene bleaching assay, and the nitric oxide scavenging method [61].

Lemongrass has noteworthy reducing activity, as its inhibition constant values were significant in the DPPH and nitric oxide methods. Essential oil of lemongrass (with an origin in Egypt) has an inhibition constant 50 value of 1.0 milligram per milliliter and possesses greater antioxidant activity than lemongrass with an origin in Saudi Arabia, which demonstrated an inhibition constant of 6.9 milligrams per milliliter [62]. It has been reported that LEO found in Indonesia possesses less activity as an antioxidant in comparison to commercial essential oil and ascorbic acid [63]. Furthermore, it has been reported that LEO has a lower scavenging activity but greater FRAC (ferric reducing antioxidant capacity). Thus, it is efficient in the chelation of iron (II) ions compared to ascorbic acid and butylated hydroxytoluene [64].

The antioxidant activity of LEO is due to the synergistic actions of its active components (α-citral, β-citral, myrcene, and eugenol). Another study on the use of an ethanolic extract of lemongrass in the storage of cooked and shredded chicken breast revealed that the water activity of the product was not affected by the addition of lemongrass extract. Furthermore, it was observed that the phenolic compounds present in lemongrass delayed the lipid oxidation in the product and may prevent lipid peroxidation. The study concluded that the ethanolic lemongrass extract could be a potential and natural source of antioxidant activity [41].

Much work has been done on the antioxidant properties of the essential oil of *C. citratus*. The species found in Brazil is known to have lower antioxidant potential when using the DPPH method, but when the analysis is done with the carotene assay, the essential oil possesses noteworthy antioxidant capacities [65].

The antioxidant capacity of an essential oil varies with the distillation methods, the origin of the species, and the portion of the plant treated. Likewise, the oil from the stalk of lemongrass, which is steam distilled, is known to have greater potential as an antioxidant in comparison with the oil from the entire lemongrass plant, which is distilled by water. Moreover, the oil made from the steam-distilled leaves had a higher antioxidant potential than the water-distilled leaves [66]. The phenolic and terpenoid components may explain the redox potential by engaging in multiple viable processes, such as the donation of hydrogen ions, the activity of hunting for free radicals, the chelation of transition metals, and the quenching potential. It is important to have a combination of diverse components of oil because the quantity of each component may affect the antioxidant activity of the entire essential oil [67]. The discussion is concluded on the evidence that LEO is effective in replacing butylated hydroxytoluene in raw food packing because of the presence of eugenol, which possesses scavenging activity against free radicals. Studies show LEO is effective in preserving food products not only at the domestic but also at the industrial level due to its antioxidant potential.

### 3.4. Anti-Insecticidal Activity

Lemongrass also possesses derivatives that are known as allelochemicals which affect the biology of insects and their actions, thus making LEO an effective biopesticide [68]. The insecticidal property of LEO is attributed to the presence of bioactive acyclic and cyclic terpenes. Multiple studies reveal that lemongrass is efficient in killing insects still at the larval stage, such as cabbage looper [69], and has repulsive behavior on various insects due to the presence of caryophyllene (0.57%), germacrene D (2.24%), and caryophyllene oxide (0.58%). Although these bioactive constituents are present in a limited amount, they are effective in killing and repelling insects such as malarial vector mosquitos and common houseflies when compared with commercially prepared insecticides [70].

Essential oils are highly volatile; therefore, they enter the respiratory system of insects. Their bioactive components affect the olfactory receptors inside the respiratory system of insects [71]. LEO possesses the quality of activating the olfactory receptor neurons [72]. Essential oil, along with olfactory neurons, instigates other neurotoxic responses [73,74]. The activity of neurotransmitters, including octopamine and acetylcholine esterase, is inhibited by essential oils. Essential oils impart fumigant toxicity and cause a reduction in the synthesis rate of DNA with other sub-lethal to lethal damage [71,75].

Citral, which is a major constituent of LEO, interacts with intracellular oxygen radicals and oxidative stress, thereby controlling cell propagation [76,77]. Hormonal imbalance, membrane damage, decreased signal transduction and cytotoxicity are caused by the presence of the bioactive components of essential oil inside the host [70,75]. These various components of LEO target different multiple sites inside the insect. Thus, it has been proven effective in killing insects [78].

### 3.5. Limitations

Although essential oils are proven to be beneficial, there are some limitations in their usage for preserving food, their potential against multi-resistant microbes, the satisfactoriness of customers, and the sensory attributes of the products. The components in an essential oil that are hydrophobic get affected by the lipids, proteins, and starch present in the food [79]. With the benefits of lemongrass come the hazardous effects as well. Some studies have revealed that greater levels of lemongrass may cause problems with the senses of taste and smell, while essential oil used in lesser amounts is thought to have reliable effects on humans. It has been reported in in vitro studies that the concentration of lemongrass essential oil used in food commodities is between 0.2–10 µL/mL, which is considered 25–100 folds high and is effective in controlling [80]. Furthermore, it is reported that the excessive and casual overutilization of LEO may cause tumors, damage to genes, and cancerous effects [81]. Because of the health concerns, more research is needed to explain the effects of lemongrass. Moreover, there is a need to determine the concentration of essential oil which can exceed the safe limit dose to achieve an acceptable result in food preservation. Exceeding this safe limit can result in undesirable organoleptic changes in the product, which will make the food product undesirable for the consumer [82].

## 4. Combination of LEO with Different Coating Materials

Bio-preservatives are active substances that are present naturally or extracted from food which can prevent the degradation caused by food spoilage microorganisms and extend the shelf life of food products [83]. In these preservatives, phenolic components are present that have the ability to defend against bacteria, fungi, parasites, viruses, and insects and maintain the sensory quality of food products. However, there is a drawback in that they are not resistant to low pH, enzymes, and high temperatures, which means they can be degraded in the food matrix. This problem may be solved by the application of innovative methods of introducing preservatives in food, such as encapsulation, which can allow these molecules to remain active and be protected from changes in the environment. The material used in encapsulation serves as a carrier for the phenolic components to perform their function effectively [84]. Encapsulation can be done by introducing edible films or coating and nanoemulsions. According to many studies, this innovation is the most effective tool to enable bioactive substances to perform their function. These techniques are eco-friendly and markedly prolong the shelf life of animal-based foods, vegetables, and fruits [85,86].

### 4.1. Nanoemulsion

Nanotechnology has been given special attention due to its revolutionary effect in the food sector. This technology deals with particles with a size of approx. 1–100 nanometers that are used to produce and consume constituents that have innovative properties. To preserve food and make it free from organisms, the demand for nanoparticles has increased in the areas of processing, packaging, prolonging the shelf-life of food products, developing functional foods, and investigating pathogens present in the food. The increasing applications in the field of nanotechnology have enabled the progression of nanocarriers against microbes in the food industry [87]. Innovative methods such as nanoencapsulation overpower the limitations of the solubility of water, the physical properties, and evaporation that restrain the integration of essential oils (lemongrass) that have antioxidant and antimicrobial properties [88,89]. Due to nanoencapsulation, citral, the active component of lemongrass, works effectively in low concentrations that are enough to stop the growth of microbes and slightly alter the product quality. The activity of citral against microbes is being amplified [90].

In the food industry, being natural preservatives, essential oils and their nanoemulsions are exclusive additives, as they possess properties against microbes and free radicals. Moreover, it has been seen that nanoemulsions provide physical stability. Bioactivity has been increased by the application of nanoemulsions, which further lessens the changes that occur in the sensory properties of food products [91]. Because of this technique, LEO can be used on crops that are post-harvest. LEO can also be used to protect seeds from microbes so that the seed cannot be spoiled qualitatively and quantitatively and can be used for planting. In a study, LEO was added to make a nanoemulsion for the coating of ready-to-eat grapes. The results showed that the coating was efficient against *Salmonella* and total mesophilic aerobes, yeasts, and molds. Thus, it can prolong the life span of grapes during storage, as the coating possessed effectual properties against microbes and retained the sensory properties of the grapes [92].

An experiment has been carried out in which high-pressure homogenization was used to include LEO in a carnauba-shellac wax-based nanoemulsion. After 2 h of storage, it was revealed that the nanoemulsion effectively decreased *E. coli* and *L. monocytogenes* populations by 8.18 log CFU/g. When the nanoemulsion was loaded with LEO and applied to apples, the aerobic bacteria were lessened by 1.4 log CFU/g compared to the apples without the nanoemulsion. The coating plays an effective role against molds and yeasts. Furthermore, it has been noticed that the loading of nanoemulsions does preserve the physiochemical properties of apples and grapes. The coating also plays a vital role in the inhibition of pathogenic bacteria such as *S. typhimurium* and *E. coli* in these fruits [92,93]. Similarly, LEO-including nanoemulsions have an effective role in inhibiting the spoilage of berries by improving their life span and safety from microbes and reducing aging. The literature indicates that a value of coating of 3.0 g/100 g inhibited *E. coil* and *S. typhimurium* that are loaded on barriers by more than 2.6 and 3.2 log CFU/g, correspondingly. The glossiness and the taste of the berries were not considerably altered; in fact, this emulsion even enhanced the glossy effect of the berries. This emulsion produces pleasant effects such as maintaining firmness, antioxidant activity, decreasing weight loss, and preventing the degradation of phenolic compounds. In berries, the coating also causes a delay in increasing the total concentration of anthocyanin [94].

A nanoemulsion prepared from LEO and alginate by ultrasonication and micro-fluidization exhibited antibacterial potential against *E. coli*. However, the production process of the nanoemulsion determined its biological activity. When evaluating both preparation methods, it was revealed that ultrasonication was found to be less beneficial, while micro-fluidization showed improved activity against microbes. Nevertheless, many studies are still being conducted regarding these techniques [89]. Investigations were done against *Gaeumannomyces graminis* fungus, in which lemongrass and clove oil were encapsulated into nanoparticles made up of silica. The results showed that these nanoparticles are effective against fungus up to 3 times. However, the use of sodium alginate in the nanoparticles has better control against fungus, as explained in Table 2. The bioactive components obtained from LEO like limonene [95], eugenol [96], geraniol [97], and citral [90] are also integrated into coatings such as edible films and nanoemulsions. The nanoemulsion techniques are limited because of the comparatively elevated cost of production and scarcity of the approved materials used in production. These problems put a limitation on the usage of nanoemulsions in the food industry and also possibly hinder the use of nanocarriers incorporated with LEO.

### 4.2. Edible Films

Edible films are fine layers applied to the surface of foods or that form amid distinctive layers of compounds to avert the movement of solutes, humidity, and oxygen in food products [98]. The research on lemongrass usage has generated novelties in different fields, such as the use of an edible coating, the synergistic effect with other preservation procedures, and nanoemulsions. The work done in these fields is to certify the use of lemongrass in packaging instead of its active component in the food itself. Aesthetic appearance, hindrance against oxygen, biodegradability, and edibility determine the selection of appropriate procedures and inventions for edible films [99]. In addition to these factors, it is also important that substances bearing antimicrobial activity, nutrients, and antioxidant activity are supported by the edible coating. Edible coating brings benefits that include inhibiting the growth of pathogenic microorganisms, prolonging the rancidity of fat in meat, preventing discoloration, and improving the quality of coated food nutritionally. This can all happen when antimicrobial agents are integrated into the polymer matrix of edible coatings [100].

Hydrocolloids, composites, and lipids are the categories of edible film constituents. Sodium caseinate, soy protein, alginate, egg albumin, agar, cellulose derivatives, chitosan, whey protein, fruit puree, and starch are the polysaccharides and proteins that are included in hydrocolloids. Fatty acids, waxes, and acylglycerols are included in lipids, while the composites include both hydrocolloid and lipid constituents [98]. In one of the studies, LEO loaded on a cellulose nanofiber-polyethylene glycol composite was assessed for its antioxidant and antimicrobial activities. The results reveal that this composite is effective against *E. coli* and *S. aureus* and contain free radical scavenging activity, as explained in Table 2.

**Table 2:** Combination of LEO with different coating materials for food preservation.

Coating Material	Test/Target Component	Procedure	Result	Reference
LEO + clove oil + mesoporous silica nanoparticles	Wheat/ *G. graminis*	Lemongrass and clove oil are encapsulated into nanoparticles that are made up of silica against *G. graminis*, a problematic agent for wheat. The study was performed both in vivo and in vitro. To keep nanoparticles stable, sodium alginate is also used in in vivo studies.	It was revealed that the nanoparticles with lemongrass and clove oil were effective against this fungus up to three times. However, with sodium alginate, the nanoparticles have better control against fungus. Thus, this coating is beneficial to control wheat diseases.	[101]
LEO + cinnamon oil + gum arabic + sodium caseinate	Guava/ enzymatic rancidity	Five formulations were made to analyze the storage activity of guava. These formulations were prepared with different concentrations of LEO and cinnamon oil but with the same quantity of gum arabic and sodium caseinate. Guavas were stored and were investigated.	This coating revealed the lower activity of polyphenol oxidase and peroxidase and greater radical scavenging activity. Retention of flavonoids, ascorbic acid, and phenol content also occurred. Samples with 2% LEO formulations were good to extend the shelf life of guava up to 40 days compared to uncoated samples.	[102]
LEO + cellulose acetate	Cellulose acetate/ *E. coli* and *S. aureus*	Cellulose acetate combined with LEO made nanocapsules with the help of the solvent/anti-solvent method. The diameter of the capsules was between 95 and 185 nm.	The nano-capsules exhibited good antimicrobial properties against *E. coli* and *S. aureus.*	[103]
LEO loaded on a cellulose nanofiber-polyethylene glycol composite	Cellulose acetate/ *E. coli* and *S. aureus*	Cellulose nanofiber and polyethylene glycol composites were made through melting and mixing processes. The index for antioxidant and antimicrobial properties was assessed.	The results showed the total phenolic content, antioxidant capacity, free radical scavenging, and antibacterial activity against *E. coli* and *S. aureus*.	[104]
Lemongrass- and clove oil-based non-ionic nanoemulsion	Tomato seeds and seedlings/*Fusarium oxysporum lycopersici*	A nanoemulsion was made as an oil-in-water emulsion that contained LEO and clove oil. It was developed against *Fusarium oxysporum lycopersici* (FOL).	Results revealed the disruption of the membrane of the fungal species.This nano-formulation lessened the wilting of tomatoes that occurred due to *Fusarium* species by up to 70.6%.This emulsion, when applied to seeds and seedlings, did not show phytotoxicity and controlled the wilting disease of the tomatoes.	[105]
LEO + plant-based emulsifier	Iceberg leaves/ *Lactobacilli* or *Salmonella*	The microemulsion was formed between LEO and a plant-based emulsifier to improve the miscibility of oil in water. Lettuce was washed and inoculated with *Lactobacilli* or *Salmonella* Newport.Then, those leaves were treated with a microemulsion of 0.1%, 0.3%, or 0.5% and stored at 4 °C.	The concentration of 0.5% microemulsion was effective in lessening the browning of iceberg leaves due to *Lacticaseibacillus casei*.	[106]
LEO + β- cyclodextrin + PVA starch	Aquatic products/ *Shewanella putrefaciens*	A coating was prepared in which LEO was implanted into β-cyclodextrin by the co-precipitation method. Then PVA-starch active films were incorporated in LEO/β-CD.	The incorporation of PVA-starch films into LMO/β-CD revealed effective antioxidant activity and antimicrobial activity against *Shewanella putrefaciens*. This made it a potential agent in the packaging of aquatic products.	[58]
LEO + chitosan-based edible coating.	Eggs/shelf life	Hen eggs were coated with LEO at different concentrations (0.2%, 0.3%, 0.4% and 0.5%) and with 1.5 % chitosan. Progress was measured at 8 weeks to assess the lemongrass essential oil concentration.	LEO with 0.4% concentration and a 1.5% chitosan edible coating was effective in increasing the shelf life of hen eggs for 8 weeks. It was an effective method to preserve eggs at room temperature.	[107]
LEO + alginate-based film	Trehalose, capsule and Tween/ *E. coli*	An encapsulating agent, such as trehalose, capsule, and tween 20, was used with LEO. This was further incorporated into 1% *w*/*w* alginate and 1% *w*/*w* sorbitol.	This experiment revealed that the microencapsulation was effective against microbes.	[108]
LEO microcapsules + alginate-based film	Fresh fatty foods/ *E. coli* and *L. monocytogenes*	LEO microcapsules were prepared by the emulsification-separation method using sodium caseinate as wall material. The film-forming solution was prepared by dispersing 1 g of sodium alginate in distilled water at 50 °C for 30–45 min, and1 g of sorbitol was then added. After cooling down the mixture to 25 °C, the microencapsulated LMO was added, and the mixture was homogenized at 5000 rpm for 5 min.	LEO with concentrations of 1250, 2500, and 5000 ppm inhibited the growth of *E. coli* and *L. monocytogenes*.	[109]
LEO + gum arabic + maize starch + glycerol	Pomegranate/ post-harvest shelf life	The study involved gum arabic maize starch with 0.5–1.5% concentration each, 2–4% LEO, and 0.5–1% glycerol. This coating was developed using RSM (response surface methodology) on “Wonderful” pomegranate fruit. After the storage, for 42 days, the % weight loss of the fruit was evaluated.	The formulation with 0.5% gum arabic, 0.5% maize starch, 3% LEO, and 1.5% glycerol was more effective in post-harvest treatment for “Wonderful” pomegranate. This coating inhibits loss of weight and is efficient in maintaining quality during storage and increasing shelf life.	[110]
LEO + chitosan-based films	Food packaging/ *Salmonella* Typhi, *E. coli, Bacillus cereus,* and *L. monocytogenes*.	In this study, 1.5 g chitosan, 100 mL of water, and acetic acid (1.5 mL) were dissolved. Then, 0.5 mL Tween 20 as an emulsifying agent and 0.5 mL glycerol as a plasticizer were added.In this solution, 1, 3, 5, 7, and 9% concentrations of LEO were used.	The integration of 9% LEO in chitosan film was the most efficient (*p* < 0.05) against *Salmonella* Typhi, *E. coli, Bacillus cereus,* and *L. monocytogenes*.This acts as a potential source for antimicrobial food packaging.	[111]
LEO + flaxseed meal protein (FMP) film	*L. monocytogenes* and *E. coli*	FMP film was formed with 5g FMP, 2 g fructose, and 0.03 g ferulic acid. LEO with 0.5, 1.0, 1.5 g and 0.25 g of Tween 80 were added to this film to have antimicrobial activity.	The study showed a reduction in counts of *L. monocytogenes* and *E. coli* when wrapping pen shell adductor muscle treated with FMP film comprised of 1.0% LEO and stored at 4 °C for 12 days.	[47]
LEO with sodium alginate and chitosan-based coatings	Fruit (pomegranate)/ *B. cinerea* *Penicillium* spp.	The coating was prepared by adding 1 g of soluble chitosan powder dissolved in water to 1% *v*/*v* glycerol and LEO with concentrations of 1.5%, 3%, 6% *v*/*v*. Sodium alginate was prepared by adding 1% *w*/*v* sodium alginate in glycerol.	This coating was effective in reducing the decay severity of the fruit and inhibiting the germination of spores. It is effective against fungus and helps extend the shelf life of fruit.	[110]
LEO with sago starch as edible film	Overall anti-microbial effect studied	The coating was prepared by adding starch (3% to 10% *w*/*v*), and lemongrass oil (1%, 5% *v*/*v*). The effects on tensile strength, water vapor permeability, and antimicrobial activity were evaluated.	The results of the study showed that 4% sago starch and 5% *v*/*v* LEO made an effective edible film against bacterial growth and water vapor permeability.	[112]
LEO + chitosan emulsion	*S. typhimurium*, total mesophilic aerobes, molds, and yeasts	LEO was homogenized with a chitosan solution and Tween 20 by dynamic high-pressure processing or high-shear mixing.	The results showed that dynamic high-pressure coating was most effective in reducing the growth of *S. typhimurium* and inhibiting the growth of total mesophilic aerobes, molds, and yeast. It was efficient in preserving the color and sensory attributes of the product.	[92]

The formation of edible films is an effective method for enhancing the condition and prolonging the life span of fruits by preventing the loss of moisture and the rate of respiration [113]. The edible films act as useful constituents that have activity against microbes that may help in preserving the color, aroma, and reducing scavenging free radical formation, and hence, are beneficial for the fruits [114]. In addition, an edible coating improves the aesthetic value by providing a glossy appearance. The fruit firmness, loss of weight, reduction of the occurrence of anthracnose, browning of the vascular part, and gray mush are all preserved by LEO with the reformed atmospheric packaging. Furthermore, after maturation at 25 °C and 18 days of cold storage at 10 °C, it exhibited pleasing taste, consistency, aroma, and better tolerating quality [115].

Different coatings and edible films against microbes have also been introduced to preserve meat, meat products, and fish. The conservation of sea bass slices against hydrogen sulfide-producing bacteria, *mesophilic*, *enterobacteria,* and psychrophilic microbes has been carried out by LEO 25% *w*/*w* with a coating material (*Aluterus monoceros*) [77,116]. In another study, the effect of gelatin/palm wax/LEO (GPL)-coated Kraft paper on the quality and shelf life of ground beef stored at 4^◦^C for seven days was analyzed. It was observed that after seven days, the ground beef packed with GPL-coated paper was better in quality. The LEO present in the coating delayed the lipid oxidation and microbial growth, thus lowering the pH and reducing color changes. It extended the shelf life of ground beef by four days in comparison to other treatments [117].

### 4.3. Microemulsions

To prevent the degradation of LEO microparticles, these were thermally secured. A molecular encapsulation with cyclodextrin was shown to prevent the loss of citral during spray drying. It was observed that α- and β-cyclodextrins were more effective for encapsulation [118]. The efficacy of an LEO-blend microemulsion in curry paste to demonstrate activity against bacteria and enhance stability and physical properties showed improvements by preserving and stabilizing the natural benefits of LEO. It has been revealed in the study that this LEO-blend microemulsion is highly stable, has a low acidic value, is moderately conductive, and is transparent in appearance. Therefore, to conclude, this LEO microemulsion can be a substitute for a natural preservative, as with all these traits, it carries an antibacterial effect that is due to the presence of citral, citronellol, d-limonene, citronellal [119]. Lemongrass extract in microencapsulation revealed enhanced antioxidant activity. Maltodextrin and β-cyclodextrin were used separately with lemongrass for microencapsulation. The results demonstrated that lemongrass extract with a β-cyclodextrin microcapsule had improved antioxidant activity [120].

Similarly, radio-sensitization with microemulsion in orange juice was investigated against fungi, namely *Penicillium chrysogenum, Aspergillus niger*, and *Saccharomyces cerevisiae*. Lemongrass and oregano oil with citrus extracts were included in a microemulsion that was treated with various doses of γ-irradiation. Resultantly, essential oils with the citrus extract microemulsion and irradiation by gamma rays showed a synergistic effect against fungi by increasing the radiosensitivity of fungi and causing the complete eradication of the yeast and mold in orange juice, as shown in Table 1. Instead of preservatives that are synthetically produced, natural plant extract microemulsions with gamma radiation is a significant substitute that provides food with better flavor and safety and has high consumer acceptance [40].

Furthermore, the efficacy of microcapsules against microbes was evaluated in Coelho cheese. Maltodextrin and gum arabic were used for the formation of microcapsules. Spray drying was carried out at a temperature of 170 °C and a 0.9 L/h feed flow rate for microencapsulation. The ratio between LEO and the material was 1:5 (m/m). The results showed that microencapsulated essential oil had higher thermal stability than simple essential oil. α-citral and β-citral were the major active components of the essential oil. Both the essential oil without microencapsulation and with microencapsulation showed the inhibition of coliform growth at 45 °C. It has been revealed in the study that the effectiveness of LEO remained with the microencapsulation when subjected to the cheese. This method is known to be most effective in controlling microorganisms and extending the shelf life of dairy products. The natural essential oil has effective antimicrobial activity, but it protects the food for a shorter time due to its immediate volatilization. Due to microencapsulation, the essential oil would not be degraded early and would have increased lifespan [43].

## 5. Combination of LEO with Other Essential Oils

Lemongrass worked in combination with oregano essential oil and exhibited effectiveness against microbes such as fungi. A combination of essential oils such as lemongrass and oregano are known to be efficient against *A. niger, Pseudomonas chrysogenum,* and *Saccharomyces cerevisiae* [40]. When LEO and extracts of lime peel were added to chicken patties, the antimicrobial actions and the antioxidant potential were enhanced in both extracts [121]. The combination of LEO and lemon basil oil was evaluated to extend the shelf life and prevent bacterial growth in chicken fillets. The results showed that this combination prevented microbial growth on meat for 9 days of storage and extended the shelf life by up to 6 days [39]. In short, this information, when combined, proposes that lemongrass with other essential oils is effective against multi-resistant strains. These methods either balance or improve the qualities of lemongrass. Therefore, for the preservation of vegetables, meat, seeds, fruits, and dairy, lemongrass in combination with other essential oil is efficient.

## 6. LEO with Other Treatments

A study was conducted to evaluate the effect of LEO (0.2%) and modified atmosphere packaging on the storage quality of strawberries. Strawberries are liked by consumers due to their great flavor and health benefits, but they are delicate and may degrade within 2–5 days without cold storage and preservatives. This study demonstrated that the combined effect of lemongrass and modified atmosphere packaging prevented weight loss, preserved sensory qualities, and prevented chemical and microbial spoilage. Thus, lemongrass with atmosphere packaging could be used to extend the duration of storage [113]. Another example to control post-harvest fungal spoilage was the combination of chitosan with lemongrass against Colletotrichum species on guava, mango, and papaya. The purpose of this study was to control mycelial growth and decrease anthracnose development in fruits. The results revealed that the combination has a synergistic effect in controlling the mycelial growth and decreasing the development of anthracnose lesions on fruit, as explained in Table 1 [38]. It has also been observed that the incorporated thermal LEO treatment displayed improved results compared to the separate treatments of LEO or thermal treatment. This study was carried out for the preservation of fruit juice against yeast that causes food spoilage [122]. The effect of chitosan/cassava sago-based film incorporated with LEO was evaluated for cold preservation. The results showed that it could be used as an alternative in the preservation of perishable fruits. This film reduces microbial growth and preserves the firmness of fruits, therefore extending the shelf life [123].

## 7. Commercial or Industrial Application of LEO

LEO has been recommended as safe for industrial utilization as described earlier. Likewise, many studies discussed earlier have shown its antibacterial, antifungal, and excellent preservative potentials, which allows its utilization in the food industry. Some further examples have also been listed in the European patent specification (EP1 278 420B1) entitled “Antimicrobial composition formulated with essential oils” [124] and, similarly, United States Patent No.: US 9,687,429 B2, entitled “Antimicrobial compositions containing low concentrations of botanicals” [125]. Similarly, the microbiological decomposition of bakery goods impairs quality features, results in economic loss, and also poses a health risk to consumers. The use of essential oils, especially LEO, as a product ingredient or as part of the packaging system has confirmed that bakery products have a longer microbiological shelf life, as it not only limits fungal growth but also improves oxidation stability. However, before commercial application, the possible detrimental influence of these chemicals on the sensory qualities, such as scent and taste, of the product must be explored, and future studies are invited in this regard [126].

## 8. Future Perspectives

Numerous studies have been carried out, and various other studies are ongoing in the field of food preservation. The use of essential oils is gaining interest, as natural preservatives are more beneficial compared to artificial ones. Currently, techniques such as the use of starch-based edible films, nanoparticles, and microemulsions have opened new doors for food preservation techniques. These techniques are used to preserve a wide range of food products such as meat, fruits, and vegetables. The shift toward natural preservatives complies with consumer demands and health perspectives.

## 9. Conclusions

Lemongrass has driven special attention in the food industry because of its potential against various microorganisms. Currently, many studies are being conducted related to its preservative potential. Such studies on lemongrass and its essential oil recommend its utilization in various domains of food science, especially in post-harvest techniques, strengthening storage conditions for fruits, meat products, seeds, and grains. Such application of lemongrass has resulted in prolonged safe packaging to prevent spoilage and extend the shelf life of products. LEO is less stable due to its high volatility and can be degraded at high temperatures, which nullifies its benefits in preservation. Therefore innovative technique adaptations, such as microencapsulation, nanoemulsions, and edible films, can result in better outcomes. These innovations help maintain the antimicrobial and antioxidant activity of LEO inside the food matrix without degrading food or oil and play a vital role in the extension of the shelf life of food products. Still, some concerns need to be resolved, such as the maximum safe utilization limits and exploration of the detailed role of individual active components of lemongrass.

## Figures and Tables

**Table 1:** Antimicrobial and antifungal activity of lemongrass in a food.

Component	Food Item	Investigated Microorganisms	Outcomes	Reference
LEO	Laboratory cultures	*Bacillus cereus, E. coli O157:H7, Klebsiella pneumoniae, Staphylococcus aureus* and *Candida albicans*	The extract showed antimicrobial activity against all tested foodborne pathogens, including *B. cereus*, *E. coli O157:H7*, *K. pneumoniae*, *S. aureus*, and *C. albicans* with inhibition zones of 12 mm, 7.5 mm, 11 mm, 10 mm, and 9 mm, respectively	[29]
	Fresh cabbage and radish sprouts	*E. coli O157:H7*, *B. subtilis*, *S. aureus*, *S. typhimurium*	Higher antimicrobial efficacy	[30]
	Oats	*Aspergillus flavus*, *Aspergillus parasiticus*	Strongest inhibitory effect on the mycelial growth and sporulation at a concentration of 500 µL/L	[31]
	Bread	*Penicillium expansum*	The growth of *P. expansum* was inhibited for 21 days at 20 °C with 750 L of oil/L	[32]
LEO + citrus extract+ lactic acid	Red pepper	*E. coli*	A relatively stronger inhibition effect started in the first days of storage and significantly reduced the bacterial growth from day 7	[33]
		*S. typhimurium*	A strong bactericidal effect from the first day of storage, with a total inhibition on day 5 of storage	
	Cranberries	*L. monocytogenes*	A linear strong inhibition activity from the first day of storage till day 10, and then after, a total inhibition was obtained from day 10 to the end of storage time	
		*E. coli*	An immediate inhibition effect (*p* ≤ 0.05) on day 1; on day 4, total inhibition of *E. coli*	
		*S. typhimurium*	Significantly inhibited activity from day 1, with complete inhibition on day 14	
	Pre-cut/pre-fried Potatoes	*Salmonella enterica*	Inhibitory effects (*p* ≤ 0.05)	
LEO	Salmon Cod	*L. monocytogenes* *Listeria welshimeri*	Lemongrass was effective by showing lower minimum bactericidal concentration and minimum inhibitory concentration	[34]
	Peanuts	*Aspergillus* species	Lemongrass was found to be effective in inhibiting the growth of *Aspergillus flavus*	[35]
	Yogurt	74 spoilage yeast isolates	Its antifungal activity resulted in the complete growth inhibition of *D. hansenii* and *Y. deformans* and reduced the growth of *C. pararugosa* isolates	[36]
	Camel burger	Total bacterial count *Psychrophilic* bacteria	Decreased the count of bacteria	[37]
LEO + chitosan	Guava, mango and papaya	*Colletotrichum* species (*C. asianum*, *C. siamense*, *C. fructicola*, *C. tropicale* and *C. karstii)*	Combinations of chitosan (2.5, 5 or 7.5 mg/mL) and LEO (0.15, 0.3, 0.6 or 1.25 μL/mL) inhibited the mycelial growth of all tested fungal species	[38]
Lemongrass and lemon basil essential oil	Chicken fillets	Bacterial growth	A combination of lemongrass and lemon basil essential oils at the optimal ratio of 1:1% *v*/*v* was capable of reducing bacterial growth on meat during 9 days of preservation and prolonging the shelf life of the meat for up to 6 days	[39]
1% lemongrass and oregano 1:1 and citrus extract	Orange juice	*Aspergillus niger*	Significantly reduced the concentration of *A. niger* from day 0. After 10 days of storage, total inhibition of *A. niger* was noticed	[40]
		*Pseudomonas chrysogenum*	*P. chrysogenum* in orange juice was decreased from 3.1 logs CFU/mL on day 0 to a non-detectable level on day 7	
		*Saccharomyces cerevisiae*	Significantly decreased (*p* ≤ 0.05) the population of *S. cerevisiae*	
Hydro-ethanolic extract of lemongrass	Chicken breast	*Staphylococcus, Salmonella sp, and Coliforms*	The presence of coagulase-positive *Staphylococcus, Salmonella sp,* and *Coliforms* at 45 °C during 60 days of storage were not detected	[41]
Lemongrass essential 25% *w*/*w* and gelatin extracted from the skin of unicorn leatherjacket (*Aluterus monoceros*) films	Sea bass slices	*Mesophilic* *Psychrophilic* *H_2_S* producing bacteria	The film showed activity against these microorganisms	[42]
Microencapsulated LEO	Coalho cheese	Total coliform *Thermotolerant coliforms*	Microencapsulated LEO was efficient during storage. There was a reduction in the amount of this microorganism for 21 days	[43]
		*S. aureus*	Absence of *Staphylococcus* over 21 days	
d-limonene, citronellal, citronellol geranial and neral	Curry paste	Bacterial growth	Effective against bacterial growth	[44]
